# Validation of the Disease-Specific Components of the Kidney Disease Quality of Life-36 (KDQOL-36) in Chinese Patients Undergoing Maintenance Dialysis

**DOI:** 10.1371/journal.pone.0155188

**Published:** 2016-05-05

**Authors:** Julie Y. Chen, Edmond P. H. Choi, Eric Y. F. Wan, Anca K. C. Chan, Joyce P. Y. Tsang, Karina H. Y. Chan, W. K. Lo, S. L. Lui, W. L. Chu, Cindy L. K. Lam

**Affiliations:** 1 Department of Family Medicine and Primary Care, The University of Hong Kong, 3/F, 161 Main Street, Ap Lei Chau Clinic, Ap Lei Chau, Hong Kong; 2 School of Nursing, The University of Hong Kong, 4/F, William M.W. Mong Block, 21 Sassoon Road, Pokfulam, Hong Kong; 3 Department of Medicine, The University of Hong Kong, Pokfulam, Hong Kong; 4 Renal Unit, Tung Wah Hospital, Pokfulam, Sheung Wan, Hong Kong; Postgraduate Medical Institute, INDIA

## Abstract

**Aim:**

The aim of this study was to evaluate the validity, reliability and sensitivity of the disease-specific items of the Kidney Disease Quality of Life-36 (KDQOL-36) in Chinese patients undergoing maintenance dialysis.

**Methods:**

The content validity was assessed by content validity index (CVI) in ten subjects. 356 subjects were recruited for pilot psychometric testing. The internal construct validity was assessed by corrected item-subscale total correlation. Confirmatory factor analysis (CFA) was used to confirm the factor structure. The convergent validity was assessed by Pearson’s correlation test between the disease specific subscale scores and SF-12 version 2 Health Survey (SF-12 v2) scores. The reliability was assessed by the internal consistency (Cronbach’s Alpha coefficient) and 2-week test–retest reliability (intraclass correlation coefficient (ICC)). The sensitivity was determined by performing known group comparisons by independent t-test.

**Results:**

The CVI on clarity and relevance was ≥ 0.9 for all items. Corrected item- total correlation scores were ≥0.4 for all, except an item related to problems with access site. CFA confirmed the 3-factor structure of the disease-specific component of the KDQOL-36. The correlation coefficients between the disease-specific domain scores and the SF-12 v2 physical and mental component summary scores ranged from 0.328 to 0.492. The reliability was good (Cronbach’s alpha coefficients ranged from 0.810 to 0.931, ICC ranged from 0.792 to 0.924). Only the effect subscale was sensitive in detecting differences in HRQOL between haemodialysis and peritoneal dialysis patients, with effect size = 0.68.

**Conclusion:**

The disease-specific items of the KDQOL-36 are a valid, reliable and sensitive measure to assess the health-related quality of life of Chinese patients on maintenance dialysis.

## Introduction

The number of people needing maintenance dialysis for end-stage renal disease (ESRD) worldwide is increasing at an alarming rate causing significant global and individual burden to health and wellbeing [1]. It was suggested that the incidence of ESRD will increase disproportionately in developing countries, such as mainland China due to the expanding numbers of elderly people [2]. In 2012, Hong Kong ranked 11^th^ among Asian countries in the prevalence of ESRD with patients aged 65 or older [3]. These patients require renal replacement therapy (RRT) in the form of kidney transplantation or maintenance dialysis. The vast majority of RRT patients in Hong Kong are managed in the public sector. The 2013 Renal Registry database in Hong Kong which reflects this patient population revealed that about 60% of patients on RRT receive dialysis. Of these, 76% are on peritoneal dialysis (PD) with the remainder on haemodialysis (HD) [4].

Maintenance dialysis is a rigorous and time consuming process which may have a significant effect on patients and their family members or carers [5]. Impacts include physical limitation, impairment of social functioning and psychological distress [6]. This results in poorer health-related quality of life (HRQOL) which has been linked to poorer clinical and service outcomes [7]. The goal of dialysis care is to prolong life while maintaining a patient’s quality of life. Therefore, a valid and reliable tool for measuring quality of life specific to patients with of kidney disease is needed as an outcome measure to monitor treatment effectiveness and also to help assess the value of other interventions tailored to improve patient care.

The Kidney Disease Quality of Life-36 (KDQOL-36) is a short form version of the Kidney Disease Quality of Life Questionnaire which has generic and disease specific components [8]. The Cantonese Chinese version of the KDQOL-36 was translated by MAPI and is available on RAND Health (http://www.rand.org/health/surveys_tools/kdqol.html). This version was developed through iterative forward and backward translation only without a cognitive debriefing step to confirm the content validity of the measure [personal communication]. The step is important to ensure that the translated measure is conceptually equivalent to the original version, and relevant and culturally acceptable to the target population. Pilot testing, usually in the form of cognitive debriefing interviews with ten patients from the target population is recommended to evaluate a measure for content validity [9].

Previous studies have evaluated the psychometric properties of the English [8] and Cantonese Chinese version of the KDQOL-36 [10]. Although the validity (criterion validity, convergent validity), reliability and sensitivity of the KDQOL-36 has been evaluated in Hong Kong before [10], a large proportion of the subjects (52.6% of the total sample) in that study were comprised of renal transplant patients which may influence the psychometric assessment of the instrument. As the KDQOL-36 was originally developed for dialysis patients [11], the KDQOL-36 may have some limitations when applied to transplant patients [12]. Some of the items in the instrument refer to signs and symptoms (such as fatigue, nausea, and problem with the access site) and effects (fluid restriction, dietary restriction and ability to travel) of end stage renal failure which may not be relevant to transplant patients. A qualitative study on transplant patients found that they were free of stress and anxiety related to the need to undergo dialysis, repeated blood test to assess renal function and the social isolated imposed by ESRD such as fatigue [13]. Their HRQOL improved because they think they can “get back to normal” [13]. On the other hand, the KDQOL-36 might miss the specific concerns of transplant patients such as anxiety and worries related to the side effects of immunosuppressants and graft rejection [12, 13].

To strengthen and build on the findings of previous validation study in Hong Kong [10], we sought to add new evidence on the psychometric properties of the disease-specific subscales of the KDQOL-36 which has not been previously evaluated. Our specific objective was to assess the content validity, construct validity, reliability, sensitivity and factor structure of the disease-specific subscales of the KDQOL-36 in Chinese patients undergoing maintenance dialysis.

## Methods

### Study Design and Subjects

#### Content Validation

Cognitive debriefing interviews on the kidney disease-specific scales of the KDQOL-36 were conducted with ten Chinese (Cantonese-speaking) patients on haemodialysis to assess the clarity, relevance and interpretation of each item by one of the authors (EPHC). Subjects for the cognitive debriefing interviews were recruited by convenience sampling, balanced for age, from one community-based haemodialysis center. Patients who could understand Cantonese were eligible to participate in the cognitive debriefing interviews. The subjects were asked: (i) whether they could understand the item; (ii) to interpret the items in their own words, and (iii) whether the items were relevant to their kidney disease [14]. The answers from the interviews were transcribed verbatim and an expert panel reviewed the results of the cognitive debriefing interviews. The content validity index (CVI) on clarity and relevance was assessed by examining the proportion of the dichotomous responses “yes” or “no”. Items with CVI ≥ 0.8 were considered to have good content validity [15].

#### Pilot Psychometric Testing

Subjects for pilot psychometric testing of the KDQOL-36 were patients undergoing peritoneal or hemodialysis recruited from two different settings (hospital-based renal units and community HD centres) in order to include patients with a spectrum of disease severity and socio-demographic characteristics. Patients from both groups were excluded if they were aged <18, could not understand Cantonese or refused to participate. Eligible patients were invited by a trained research assistant to take part in this study. The aims, procedures and nature of the study were explained before obtaining consent. Subjects who consented were subsequently interviewed by trained research assistants to complete the study instruments. All interviewers were required to read the questionnaire verbatim using a standardized approach. A total of 135 HD patients in hospital-based renal units, 118 HD patients in community HD centres and 103 PD patients were recruited and completed the survey between Oct 2014 and Sep 2015.

102 HD patients were randomly selected to repeat the questionnaire two-weeks after their baseline interview to assess test-retest reliability of the KDQOL-36. All interviewers administering the test-retest interviews were blinded to the results of the baseline interviews.

A sample size of 200 subjects (100 subjects from hospital-based renal units and 100 subjects from community HD centres) was planned, based on the recommendation for pilot psychometric testing [16]. A sample of 100 subjects in each group is able to detect a statistically significant difference between groups by independent t-test based on 80% power (p = 0.05, two tailed) with Cohen’s moderate effect size 0.4.

Patients gave written informed consent for the use of their data. The study was approved by the relevant institutional review boards: Hong Kong West Cluster UW 10–366 and UW 15–090, Hong Kong East Cluster HKEC-2010-096, Kowloon Central Cluster/ Kowloon East Cluster KC/KE-10-0208/ER-3, Kowloon West Cluster KW/EX/10-150 (34–17), Joint Chinese University of Hong Kong-New Territories East Cluster CRE-2011.051 and New Territories West Cluster NTWC/CREC/911/11.

### Study Instruments

#### Kidney Disease and Quality of Life-36 (KDQOL-36)

(a) Disease specific components

The KDQOL-36 comprises a generic and a disease-specific core. The disease-specific core has 3 subscales (4 items for burden of kidney disease; 12 items for symptom bother and problems and 8 items for effects of kidney disease). The domain scores are calculated by summation of the relevant item scores and transformation into a range from 0 to 100, with higher scores indicating better HRQOL [17, 18].

(b) Short Form 12 Health Survey, version 2 (SF-12 v2)

The generic core of the KDQOL-36 is the Short Form 12 Health Survey, version 1 (SF-12 v1). However, in the present study, we deliberately replaced the SF-12v1 with the SF-12 version 2 (SF-12 v2) because it outperforms the SF-12 v1 in terms of item wording, response scales and scoring algorithms, leading to better score precision [19] and is more commonly used in different patient populations in Hong Kong such as patients with lower urinary tract symptoms, prostate cancer and depression [20–22] and haemodialysis patients [23, 24] because it has been well validated for use in Hong Kong [25]. The population norm of the SF-12 v2 has also been established for Chinese adults in Hong Kong [25, 26]. The SF-12 v2 can be summarized into physical and mental component summary scores (PCS and MCS), with higher scores indicating better HRQOL. In this study, the SF-12 v2 was used to assess convergent validity [14, 27].

### Statistical Analysis

Since the generic core part (SF-12 v2) has been well developed and validated, only the analysis of the three subscales in the disease-specific components (burden, symptom and effects) were conducted in the present study.

Descriptive statistics were used to show the socio-demographic comparison between HD and PD group. Differences in characteristics between groups were tested using independent t-test for continuous variables or chi-square for categorical variables. Descriptive statistics, including percentage of floor and ceiling for each domain were calculated with 15% used as the threshold for a significant floor or ceiling effect [28].

The internal construct validity of disease specific domains was assessed by using corrected item scale correlation using cut-off scores ≥ 0.4 to indicate adequate correlation [29].

Factor structure was evaluated using confirmatory factor analysis (CFA). Two models were established for each disease specific domain. Several indicators such as the root mean square error of approximation (RMSEA), the comparative fit index (CFI), Tucker-Lewis index (TLI), standardized root mean square residual (SRMR) and coefficient of determination (CD) were used to assess the goodness of fit for the models. RMSEA value of <0.5, 0.5–0.1 and >0.1 indicated good, moderate and bad fit of the model to the covariance matrix, respectively [30].

The convergent validity of the KDQOL-36 was assessed using Pearson’s correlation test against the SF-12 v2 MCS and PCS, and disease specific domains. Correlation coefficients were interpreted as negligible for coefficient = 0, small = 0.1, medium = 0.3, large = 0.5, very large = 0.75.

In term of reliability, Cronbach’s alpha using cut-off scores ≥ 0.7 was used to evaluate the internal consistency of each disease specific domains of KDQOL-36 [31]. Intra-class correlation coefficient (ICC) and paired t-test were performed to assess the test-retest reliability. ICC ≥ 0.7 was considered to indicate good reproducibility [28].

Sensitivity of the KDQOL-36 was determined by performing known group comparisons for each disease specific domain. Independent t-tests were used to assess the differences in mean domain scores between HD and PD groups. The Cohen’s d effect sizes were also calculated.

All significance tests were two-tailed and those with a p-value less than 0.05 were considered statistically significant. The statistical analysis was performed in STATA Version 13.0.

### Ethics

All authors declare they have no conflict of interest

The study protocol was approved by the institutional review boards.

All procedures performed in studies involving human participants were in accordance with the ethical standards of the institutional and/or national research committee and with the 1964 Helsinki declaration and its later amendments or comparable ethical standards.

Informed consent: Written informed consent was obtained from all individual participants included in the study.

## Results

### Content Validity

Ten subjects were recruited for cognitive debriefing interviews: six males, four females; age range 41–64 years, mean 49.3 years (SD: 7.8). One out of ten subjects could not interpret the item “*problems with your access site*” and “*your personal appearance*”, respectively. The CVI on clarity and relevance achieved the 0.8 standard for all items. The results are shown in Table 1.

**Table 1:** Results of cognitive debriefing interviews.

	Content validity index		Number of subject who correctly interpreted the item
	Clarity	Relevance	
**Burden of kidney disease**			
My kidney disease interferes too much with my life	1	1	10
Too much of my time is spent dealing with my kidney disease	1	1	10
I feel frustrated dealing with my kidney disease	1	1	10
I feel like a burden on my family	1	1	10
**Symptoms and problems**			
Soreness in your muscles	1	1	10
Chest pain	1	1	10
Cramps	1	1	10
Itchy skin	1	1	10
Dry skin	1	1	10
Shortness of breath	1	1	10
Faintness or dizziness	1	1	10
Lack of appetite	1	1	10
Washed out or drained	1	1	10
Numbness in hands or feet	1	1	10
Nausea or upset stomach	1	1	10
Problems with your access site?	0.9	0.9	9
**Effects of kidney disease**			
Fluid restriction	1	1	10
Dietary restriction	1	1	10
Your ability to work around the house	1	1	10
Your ability to travel	1	1	10
Being dependent on doctors and other medical staff	1	1	10
Stress or worries caused by kidney disease	1	1	10
Your sex life	1	1	10
Your personal appearance	0.9	0.9	9

### Descriptive Statistics

356 subjects were recruited for analysis. Table 2 shows the socio-demographic comparison between HD and PD patients. PD patients were older (63.14 vs 56.62) and a larger proportion were married (71.84% vs. 59.20%) and had higher personal income (9.78% vs. 2.59% had monthly personal income ≥ $20,000) than HD patients. The ceiling and floor percentages of disease specific domains are displayed in Table 3. No floor and ceiling effect was detected for the three subscale scores.

**Table 2:** Socio-demographic characteristics of HD and PD patients.

	HD patients (n = 253)	PD patients (n = 103)	P-value
Sex			0.986
Female	61.26% (155)	61.17% (63)	
Male	38.74% (98)	38.83% (40)	
Age (mean±SD), year	56.62±12.14 (253)	63.14±12.76 (103)	<0.001*
Educational level			0.801
No formal education/Primary	36.36% (92)	34.95% (36)	
Secondary/Tertiary	63.64% (161)	65.05% (67)	
Marital status			0.025*
Not married	40.80% (102)	28.16% (29)	
Married	59.20% (148)	71.84% (74)	
Smoking status			0.893
Non-smoker	65.61% (166)	66.02% (68)	
Current smoker	4.74% (12)	5.83% (6)	
Ex-smoker	29.64% (75)	28.16% (29)	
Working status			0.365
Not working	84.52% (213)	80.58% (83)	
Working	15.48% (39)	19.42% (20)	
Monthly household income			0.186
< $20,000	78.16% (136)	69.84% (44)	
≥ $20,000	21.84% (38)	30.16% (19)	
Monthly personal income			0.005*
< $20,000	97.41% (226)	90.22% (83)	
≥ $20,000	2.59% (6)	9.78% (9)	

HD = Haemodialysis; PD = Peritoneal Dialysis

* Significant at 5% level by chi-square test or independent t-test, as appropriate

**Table 3:** Descriptive, ceiling and floor effects of disease-specific domains of KDQOL-36.

		All HD and PD patients (n = 356)		
	Mean±SD	Corrected item-total correlations	Floor	Ceiling
**Burden Score**	33.73±24.65		10.11%	1.12%
Interfered by kidney disease	23.38±28.81	0.623		
Too much time spent on kidney disease	25.21±30.11	0.458		
Frustrated	46.63±37.05	0.482		
Burden on family	39.68±37.40	0.481		
**Symptom Score**	73.83±17.62		0.28%	5.90%
Soreness in muscles	69.94±32.16	0.543		
Chest pain	85.53±25.82	0.471		
Cramps	71.35±32.35	0.415		
Itchy skin	57.65±35.10	0.520		
Dry skin	59.20±32.20	0.545		
Shortness of breath	79.92±27.90	0.543		
Faintness/Dizziness	79.56±26.64	0.522		
Lack of appetite	80.27±26.91	0.467		
Washed out/Drained	58.57±32.56	0.642		
Numbness in hands/feet	76.62±30.34	0.521		
Nausea/Upset stomach	82.44±25.68	0.451		
Problems with access site	84.97±25.01	0.291		
**Effects Score**	66.39±22.73		0.28%	7.87%
Fluid restriction	59.90±37.18	0.535		
Dietary restriction	65.17±33.01	0.562		
Ability to work around the house	69.73±32.75	0.594		
Ability to travel	46.21±38.68	0.662		
Dependent on doctor and other medical staff	77.33±27.91	0.607		
Stress/Worries	63.55±29.68	0.585		
Sex life	77.36±34.66	0.482		
Personal appearance	76.41±29.84	0.599		

HD = Haemodialysis; PD = Peritoneal Dialysis

### Internal Construct Validity

The corrected item-scale correlations for disease specific domains are shown in Table 3. Based on the standard of correlation coefficients ≥ 0.4 between items and subscales, all correlations reached the target standard except on the item related to problems with access site (0.291), demonstrating item-internal consistency.

### Factor structure

Table 4 displays the goodness-of-fit indices of the confirmatory factor analysis models for disease specific domains. The factor loadings of all items, except the one related to the problems with access site (0.319), were positive and exceeded the threshold of 0.4, indicating substantial interpretability of underlying factor structure. The model had a RMSEA ≤ 0.1, CFI ≥ 0.8, SRMR ≤ 0.1 and CD ≥ 0.95, which exceeded the cutoff of the individual fit statistics, indicating a good fit.

**Table 4:** Confirmatory factor analysis of the disease-specific domain of KDQOL-36.

	Factor Loading	95% CI
	Burden Score	
Interfered by kidney disease	0.728*	(0.638,0.818)
Too much time spent on kidney disease	0.607*	(0.502,0.712)
Frustrated	0.683*	(0.589,0.776)
Burden on family	0.623*	(0.523,0.722)
	Symptom Score	
Soreness in muscles	0.565*	(0.465,0.666)
Chest pain	0.472*	(0.361,0.584)
Cramps	0.468*	(0.358,0.579)
Itchy skin	0.539*	(0.435,0.643)
Dry skin	0.578*	(0.478,0.677)
Shortness of breath	0.580*	(0.483,0.677)
Faintness/Dizziness	0.556*	(0.455,0.656)
Lack of appetite	0.521*	(0.416,0.626)
Washed out/Drained	0.727*	(0.654,0.801)
Numbness in hands/feet	0.548*	(0.447,0.650)
Nausea/Upset stomach	0.538*	(0.436,0.641)
Problems with access site	0.319*	(0.194,0.445)
	Effects Score	
Fluid restriction	0.573*	(0.476,0.671)
Dietary restriction	0.605*	(0.512,0.698)
Ability to work around the house	0.710*	(0.635,0.786)
Ability to travel	0.699*	(0.621,0.776)
Dependent on doctor and other medical staff	0.660*	(0.576,0.744)
Stress/Worries	0.696*	(0.618,0.774)
Sex life	0.489*	(0.379,0.598)
Personal appearance	0.635*	(0.547,0.723)
RMSEA	0.080	
Pclose	0.000	
CFI	0.803	
TLI	0.782	
SRMR	0.069	
CD	0.987	

CI = Confidence Interval; RMSEA = Root Mean Squared Error of Approximation; CFI = Comparative Fit Index; TLI = Tucker-Lewis Index; SRMR = Standardized Root Mean Square Residual; CD = Coefficient of Determination

* Significant at 5% level (P < 0.05)

### Convergent Validity

Pearson’s coefficients of correlation between generic and disease specific domains are calculated and displayed in Table 5. The disease-specific domain scores had a moderate correlation with the SF-12 v2 MCS and PCS cores, supporting the convergent validity.

**Table 5:** Pearson's correlations of generic and disease specific domains of KDQOL-36.

	All HD and PD patients (n = 356)				
	PCS	MCS	Burden Score	Symptom Score	Effects Score
**PCS**	1	-0.032	0.328*	0.479*	0.415*
**MCS**		1	0.422*	0.492*	0.377*
**Burden Score**			1	0.479*	0.511*
**Symptom Score**				1	0.602*
**Effects Score**					1

HD = Haemodialysis; PD = Peritoneal Dialysis; PCS = Physical Component Summary; MCS = Mental Component Summary

* Significant at 5% level

### Reliability

Table 6 outlines the reliability coefficients of disease specific domains. A total of 102 HD subjects completed the 2-week retest questionnaires and their three subscale scores at baseline and 2-week retest were compared. The mean test-retest scores differences were all not statistically significant except that of burden score, which was just marginally significant. Moreover, good agreement was found in all subscales according to the intra-class correlation coefficients (0.792–0.924). The Cronbach’s alpha coefficients of the three subscales ranged from 0.810 to 0.931, which indicated acceptable internal consistency.

**Table 6:** Reliability of disease specific domains of KDQOL-36.

	Test and Retest (n = 102)				
	Baseline	2-week Retest	P-value	ICC	Cronbach's Alpha
**Burden Score**	37.01±22.66	33.27±22.89	0.047*	0.792	0.810
**Symptom Score**	77.49±16.42	76.63±15.14	0.522	0.835	0.889
**Effects Score**	63.16±23.62	62.86±22.85	0.831	0.924	0.931

PCS = Physical Component Summary; MCS = Mental Component Summary; ICC = Intraclass Correlation Coefficient

* Significant difference (P<0.05) on score between baseline and 2-week retest by paired t-test

### Sensitivity

Table 7 presents the comparison of disease specific domains between HD and PD patients. In general, PD patients had higher subscale scores than HD patients. The difference reached statistical significant for the effects score, which had an effect size of 0.68.

**Table 7:** Comparison of disease specific domains of KDQOL-36 between HD patients and PD patients.

	HD patients (n = 253)	PD patients (n = 103)	Effect size	P-value
**Burden Score**	33.08±22.98	35.32±28.40	0.09	0.438
**Symptom Score**	72.68±17.25	76.66±18.25	0.22	0.053
**Effects Score**	62.19±22.55	76.71±19.78	0.68	<0.001*

PCS = Physical Component Summary; MCS = Mental Component Summary; HD = Haemodialysis; PD = Peritoneal Dialysis

* Significant at 5% level by independent t-test

## Discussion

The results of the cognitive debriefing interviews on the kidney disease-specific scales of the KDQOL-36 were reassuring. The CVI on clarity and relevance reached target standards. Most people could understand and correctly interpret all the items of the KDQOL-36 and all items were relevant to them. As a content validation of this instrument has never been previously performed, our findings are the first to demonstrate that the instrument is linguistically and culturally relevant to Cantonese-speaking people in Hong Kong.

No significant floor and ceiling effects were identified in the kidney disease-specific scales of the KDQOL-36. This is different from the findings of a previous study in the US which observed a significant floor effect in the ‘burden of kidney disease’ and ‘effects of kidney disease’ subscales in Hispanic and White subjects [32]. The difference in findings reaffirmed the need for psychometric testing when adapting patient-reported outcome measures for use in different linguistic settings or among patients of different cultural backgrounds. The influence of engrained cultural and health beliefs, and disease profiles of study subjects might affect the results of psychometric testing. The lack of floor and ceiling effects indicates that the scales can potentially capture deterioration or improvement in disease-specific HRQOL along the disease trajectory.

All correlations in the corrected item-total correlation testing of the adapted KDQOL-36 reached the 0.4 standard, except the item “*problems with access site*”. There are some possible explanations. First the item “*problems with access site*” is measuring a related but slightly different domain than the other items of the subscale which relate to physical symptoms. Second, it is possible that some subjects may not have understood the item, resulting in a suboptimal result. In addition, the corrected item-total correlations observed in the present study were substantially lower than those observed in a validation study in Singapore, in which all correlation coefficients were >0.7 [33]. This may be due to the use of item-total correlation testing without correction in the Singapore study which would bias the results because the total score includes the contribution of the item. We used item and total scale score correlation corrected for overlap to assess internal construct validity [34]. This method is more stringent which may account for the lower correlations.

Contrary to the previous studies which found no significant correlation between the SF-36 PCS score and effects of kidney disease score in Hong Kong [10], and weak correlation between the SF-12 PCS and burden of kidney disease score in Singapore [35], we found that all disease-specific scores of the KDQOL-36 were moderately correlated with the SF-12 v2 PCS and MCS scores, which confirmed that their constructs are related but not equivalent. As the SF-12 v2 assesses generic HRQOL, the constructs of such measures might not be specific and sensitive enough to capture the impacts of ESRD on HRQOL. Generic measures may contain constructs of less relevance to ESRD patients and miss specific concerns of ESRD patients. In contrast, the disease-specific components of the KDQOL-36 focus on the impacts of ESRD on HRQOL, so the constructs of the disease-specific components should be more relevant to those with ESRD. Our results supported the clinical importance of the disease-specific components of the KDQOL-36.

In line with the previous study in Singapore [35], our data fit the original 3-factor model (symptoms, burden and effects). The factor loading scores of all items of disease-specific domains exceeded 0.4, except for the item related to the access site. This finding was consistent to that found in our internal construct validity assessment.

The KDQOL-36 was found to be a reliable instrument. The internal consistency (Cronbach’s alpha > 0.8) observed in the present study was good and comparable to that observed in other populations such as Mainland China [36], Thailand [37] and the US [32]. The internal consistency of this instrument was much higher than that found in a similar study previously conducted in Hong Kong [8] which may be due to our larger sample size. A sample size of at least 200 is needed to determine adequately precise reliability coefficients [38]. The 2-week test-retest reliability of the adapted KDQOL was acceptable and comparable to those observed in previous studies [10, 36, 37].

The ‘effects of kidney disease’ subscale of the KDQOL-36 was sensitive enough to differentiate between HD patients and PD patients. We found that HD patients had poorer HRQOL related to the effects of kidney disease. A previous study in Singapore also found that HD patients had poorer HRQOL than PD patients as measured by the General Health Questionnaire-28 [39]. A large difference in HRQOL related to the effects of kidney disease (effect size >0.6) between these groups was observed in the present study. This is expected as HD patients spend a significant proportion of their time bound to a dialysis machine in a health care facility with previous studies suggesting that the HRQOL of HD patients was particularly affected by “loss of freedom”, “dependence on the caregivers”, “disrupted marital, family and social life” and “fluid and dietary restriction” [40].

### Limitations

First, subjects in the present study were only recruited in government-funded dialysis settings. Patients who receive dialysis in private settings, who comprise about 5–10% of this patient population, were not represented and as their treatment experience may differ and thereby affect quality of life, the study results may be biased. Second, although the instrument is more commonly used as a self-administered questionnaire, we deliberately chose interviewer-administration because the pilot testing found that our subjects prefer interviewer-administration to self-administration. Further study is needed to assess whether the mode of administration will affect a response to the instruments. Third, only HD subjects were included in the test-retest analysis, because HD subjects go to the hemodialysis centre frequently and it was not feasible to follow up with PD subjects after 2-weeks.

## Conclusion

The disease-specific components of the KDQOL-36 demonstrated satisfactory content validity, construct validity, reliability and sensitivity in Cantonese speaking patients on peritoneal dialysis or haemodialysis. Our findings support the use of this instrument to evaluate the HRQOL of Chinese patients on dialysis although future work is recommended to evaluate the responsiveness of the instrument.
